# Non-syndromic multiple supernumerary premolars: Clinicoradiographic report of five cases

**DOI:** 10.15171/joddd.2017.009

**Published:** 2017-03-15

**Authors:** Renu Tanwar, Vipul Jaitly, Aadya Sharma, Rashmi Heralgi, Munish Ghangas, Ankur Bhagat

**Affiliations:** ^1^Department of Oral Medicine and Radiology, Faculty of Dental Sciences, SGT University, India; ^2^Department of Oral Medicine and Radiology, Faculty of Dental Sciences, ESIC Dental College, India; ^3^Department of Oral and Maxillofacial Surgery, Faculty of Dental Sciences, SGT University, India; ^4^Department of Periodontics, Faculty of Dental Sciences, SGT University, India

**Keywords:** Para-premolars, supernumerary teeth, supplemental teeth

## Abstract

Hyperdontia or supernumerary teeth in both arches without any syndromic manifestation are extremely rare. Supernumerary teeth are commonly associated with Gardner’s syndrome, cleft lip and palate, cleidocranial dysplasia and trichorhinopha-langeal syndrome. Five cases of non-syndromic multiple premolars of maxillary and mandibular arches in Indian patients are presented here. This case series reports three cases with multiple (9 in maximum), bilaterally impacted and erupted supernumerary teeth and two cases with supernumerary premolars in non-syndromic cases from Indian patients. Supernumerary teeth can be present in any region of the oral cavity. Although the occurrence of maxillary para-premolars is rare, radio-logical investigations play a major and decisive role in determining the management of such cases.

## Introduction

Supernumerary teeth are defined as the presence of extra teeth in relation to normal primary or permanent dentition. Development of multiple impacted or erupted supernumerary teeth is rare and most commonly associated with syndromes or developmental anomalies such as Gardner’s syndrome, cleft lip and palate and cleidocranial dysplasia. The prevalence of impacted supernumerary teeth in Indian population is reported to be within a range of 0.1‒1.2%.^1,2^ Case series with five cases of supernumerary teeth in relation to the premolars in Indian middle-aged patients are presented here.

## Case reports

### Case I

**Figure 1.**
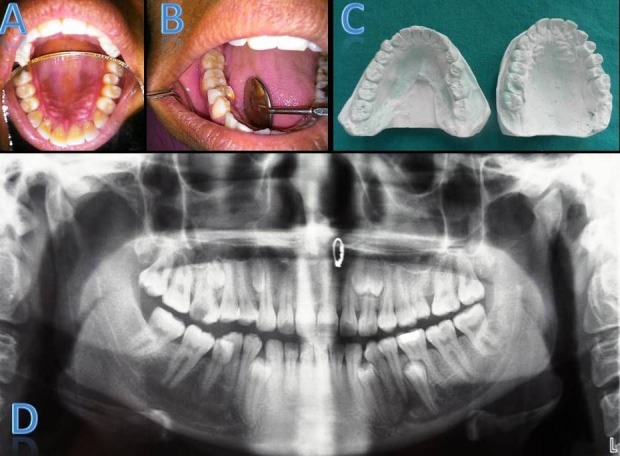
(A) Maxillary arch showing palatally placed supernumerary #15. (B) Mandibular arch showing lingually placed supernumerary #45. (C) Panoramic radiograph revealing multiple (9 in number), bilaterally impacted (7 in number) and erupted (2 in number) supernumerary premolar teeth. (D) Maxillary and mandibular study casts showing lingually erupted supernumerary #45 and palatally erupted supernumerary #15.

A 35-year-old female patient presented with a chief complaint of pain in the posterior upper left region for the past few days. Medical and familial history and extraoral examination were non-contributory. On intraoral examination, a carious lesion was present in #16 and the tooth was non-tender on palpation. Maxillary arch showed palatally erupted supernumerary tooth in the region of #25 and mandibular arch showed lingually erupted supernumerary in the region of #45 (Figure 1A,B). A panoramic radiograph revealed multiple (9 in number) impacted and erupted supernumerary premolars in maxillary and mandibular arches (Figure 1D). In the right maxillary premolar region, 2 impacted supernumerary teeth were present as interradicular radiopacity in the region of #13/#14 and #15/#16, respectively. In the left maxillary premolar region, 2 impacted supernumerary fused teeth were present as interradicular radiopacity in region of #22/#23 and erupted supernumerary in the region of #25. In the right mandibular premolar region, erupted supernumerary teeth were present interdentally in the region of #45/#46 and impacted supernumerary in the region of #43/#44, respectively (Figure 1D).). Study models were prepared, which demonstrated lingually erupted supernumerary #45 and palatally erupted supernumerary #25 (Figure 1C).) .

### Case II

**Figure 2.**
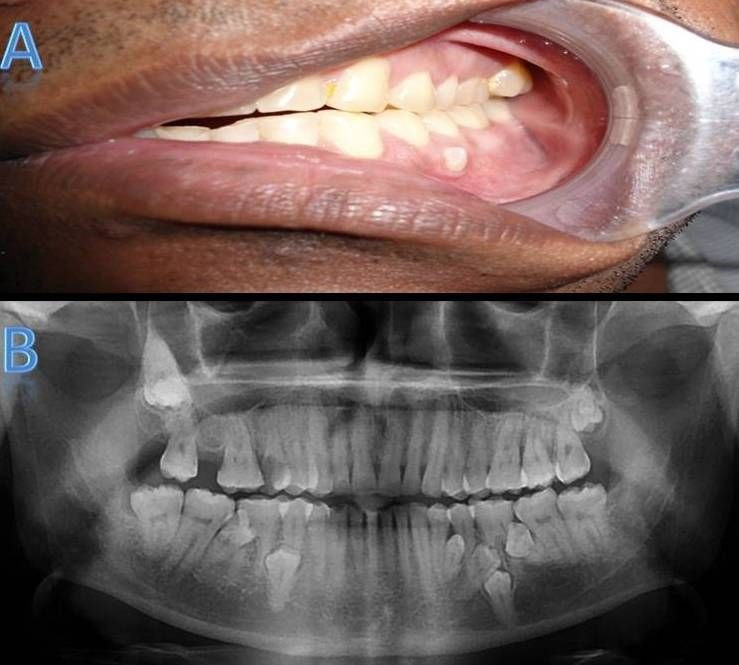
(A) Mandibular arch showing buccally placed supernumerary #35. (B) Panoramic radiograph revealing multiple (7 in number) bilaterally impacted (5 in number) and erupted (2 in number) supernumerary premolar teeth and maxillary third molar.

A 25-year-old male patient presented with a chief complaint of pain in the posterior upper left region for the past few days. Familial history and extraoral findings were non-contributory. On intraoral examination, a carious lesion was present in #15 and the tooth was non-tender on palpation. Maxillary arch showed buccally erupted supernumerary tooth #28 and mandibular arch showed lingually erupted supernumerary #45 (Figure 2A ). Panoramic radiograph revealed multiple (7 in number) impacted and erupted supernumerary premolars in the mandibular arch (Figure 2B). In the left maxillary molar region, erupted para-premolar supernumerary teeth were present. In the right mandibular premolar region, erupted supernumerary teeth were present interdentally at #45/#46 and impacted supernumerary at #44/#45, respectively (Figure 2B). In the eft mandibular premolar region, erupted supernumerary teeth were present interdentally at #34/#35 and 3 impacted supernumerary teeth at #34/#35, respectively, as interradicular teeth.

### Case III

**Figure 3.**
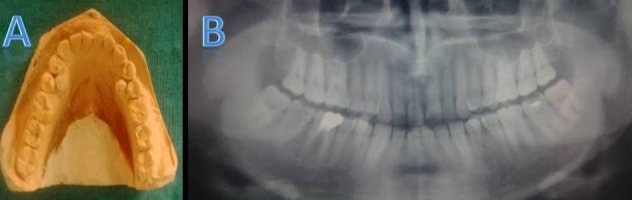
(A) Study cast showing lingually erupted supernumerary teeth #34 and #44. (B) Panoramic radiograph revealing interdental radiopacity at #34/#44 as supernumerary premolars.

A 33-year-old male patient presented with pain in the posterior lower left region since for the two months. Familial history and extraoral findings were non-contributory. On intraoral examination, two lingually erupted supernumerary premolars were observed with respect to #34/#44 region. A panoramic radiograph revealed Ellis class III fracture of #21, endodontically treated #46 and deep dental caries with respect to #37; lingually erupted supernumerary premolars were seen as radiopaque shadows between #34/#35 and #44/#45 (Figures 3A,B).

### Case IV

**Figure 4.**
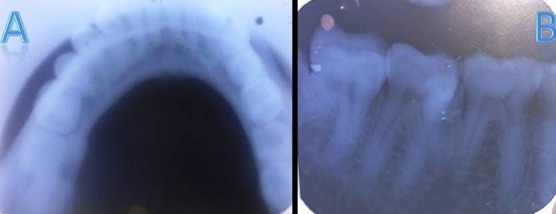
(A) Occlusal radiograph revealing well-defined solitary radiopacity along the alveolar region lingually at #36/#37, suggestive of supernumerary premolars #36. (B) Intraoral periapical radiograph showing well-defined solitary radiopacity interdentally at #36/#37.

A 28-year-old male patient presented for routine dental examination. Familial history and extraoral findings were non-contributory. Intraoral examination revealed a lingually erupted supernumerary premolar with respect to #36/#37. An intraoral periapical radiograph of the left mandibular region showed interdentally erupted supernumerary premolar at #36/#37 (Figures 4A,B).

### Case V

**Figure 5.**
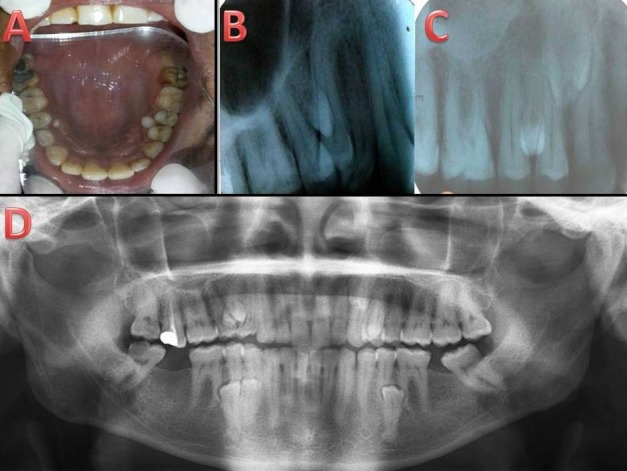
(A) Maxillary arch showing palatally placed supernumerary #14 and #15. (B) Intraoral periapical radiograph showing well-defined solitary radiopacity interdentally at #14/#15. (C) Intraoral periapical radiograph showing well-defined solitary radiopacity interdentally at #24/#25. (D) Panoramic radiograph revealing multiple (9 in number), bilaterally impacted (7 in number) and erupted (2 in number) supernumerary premolar teeth.

A 30-year-old male patient presented for routine dental examination Familial history and extraoral findings were non-contributory and intraoral examination revealed a palatally erupted supernumerary premolar with respect to #14/#24. (Figure 5A). An intraoral periapical radiograph of the maxillary left and right posterior regions and a panoramic radiograph revealed endodontically treated #16 and 2 supernumerary premolars as well-defined radiopacities interdentally between #14 and #15, 2 supernumerary premolars interdentally between #23 and #24 and #24 and #25 as well-defined radiopacities, 2 supernumerary premolars interdentally between #43 and #44 and 1 supernumerary premolar between #34 and #35 as well-defined radiopacities (Figures 5B,5Cand5D).

On extraoral examination in all five cases, there were no signs or symptoms of the presence of syndromic manifestations (Table 2). Based on clinical and radiographic findings, the cases were diagnosed as non-syndromic multiple erupted and impacted supernumerary teeth in relation to premolars (Table 1). Since the patients were completely asymptomatic, they were informed about supernumerary teeth and advised to be on regular follow-up.

**Table 2:** Clinical and radiographic features in syndromes associated with multiple supernumerary teeth

**Syndrome**	**Clinical features**		**Radiographic features**
	**Extraoral**	**Intraoral**	
**Cleidocranial dysplasia** ^10^	Partial or complete absence of the clavicles, late closure of fontanels, presence of open skull sutures and multiple wormian bones, Broad and depressed nasal bridge, Narrow high arched palate, absent paranasal sinuses due to underdeveloped maxilla, brachycephaly, hypertelorism, Bossing of the frontal, occipital and parietal regions give the skull a large globular shape with a small face **(Arnold head).**	Multiple supernumerary teeth	**Skull radiograph**: Wide-open sutures, patent fontanels, presence of wormian bones and delayed ossification of skull, decreased pneumatization of paranasal, frontal and mastoid sinuses, impacted supernumerary teeth. **Chest X-ray** shows cone shaped thorax with narrow upper thoracic diameter, absent or hypoplastic clavicles and scapulae.
**Gardner’s syndrome** ^11^	Multiple epidermoid cysts, Desmoid tumors in the skin of the anterior abdominal wall or intra-abdominally, multiple colonic polyps (familial adenomatous polyposis coli - FAP).	Supernumerary teeth, compound odontomas, hypodontia, abnormal tooth morphology and impacted or unerupted teeth, multiple peripheral osteomas, or endostoses in mandible.	**Skull radiograph:** Osteomas and odontomas in the paranasal sinuses and mandible, particularly in the angulus and corpus regions as radiopaque masses, multiple impacted teeth.
**Trichorhinophalangeal syndrome** ^12^	Cranio-facial abnormalities include a bulbous pear-shaped nose, a long philtrum, a thin upper lip and maxillary prognathism with mandibular hypoplasia, receding chin with prominent mento labial groove, large, and laterally protruding ears. Skin appendages are clinically characterized by fine, brittle, sparse, slowly growing, usually light colored scalp hair with high frontal hairline, poorly developed eyelashes at the lateral portions, brittle, and fragile slow growing finger and toe nails. Progressive osteoarticular changes and degenerative hip disease	Multiple erupted supernumerary teeth	Cone-shaped epiphyses, predominantly at the middle phalanges, Flattening of the capital femoral epiphyses, partial syndactyly, scoliosis, kyphosis, winged scapula,

**Table 1:** Clinical and radiologic features of supernumerary premolars

**No.**	**No. of premolars**	**Age gender**	**Max L**	**Max R**	**Mand L**	**Mand R**	**Bil/Uni**	**Imp/Erup**	**Treatment**
**1**	9	35/F	2	2	3	2	Bil	7/2	Extraction/ Observation
**2**	6	25/M			4	2	Bil	5/1	Extraction/ Observation
**3**	2	33/M			1	1	Bil	0/2	Extraction/ Observation
**4**	1	28/M				1	Uni	0/1	Extraction/ Observation
**5**	7	30/M	2	2	2	1	BIL	5/2	Extraction/ Observation

F: Female, M: Male, L: Left, R: Right, Max: Maxillary, Mand: Mandibular, Bil: Bilateral, Uni: Unilateral, Imp: Impacted, Eru: Erupted

## Discussion

Supernumerary teeth can be present in any region of the jaw as impacted, erupted or partially erupted teeth. Supernumerary teeth may occur as single (76–86%), double (12–73%) or multiple (<1%), unilateral or bilateral, and in one or both jaws.^3^ Presence of multiple supernumerary teeth not associated with any syndrome is a rare manifestation. In a review presented by Yousoz et al (1990), it was concluded that in cases of non-syndromic multiple supernumerary teeth, mandibular premolar region is the most affected site followed by molars and anterior region.^3,4^Supernumerary premolars are present in 8% of all supernumerary teeth with the prevalence ranging from 0.64% to 1.7%. The occurrence of non-syndromic multiple supernumerary premolars is more common in the mandible than in the maxilla (8‒10 times more prevalent), in males than in females (3 times more prevalent), in permanent dentition than in primary dentition and unilaterally than bilaterally.^3,5,6^ In case I here, multiple (9 in number) supernumerary premolars were present in permanent dentition in asymptomatic middle-aged female patients, affecting both jaws bilaterally; in case II multiple (6 in number) supernumerary teeth were present; and in case V multiple (7 in number) supernumerary premolars were present. In all three above-mentioned cases, more supernumerary premolars were impacted than erupted. In cases III and IV, lingually erupted supernumerary premolars were seen. Piattelli et al (1995) reported a similar case in a 22-year-old male patient with five supernumerary impacted and erupted premolars in the mandibular region.^7^

The exact etiology of formation of supernumerary teeth is not clear, though various hypotheses have been proposed. These include phylogenetic theory of atavism, dichotomy theory (division of single tooth bud into two homologous or heterogeneous parts), hereditary (an autosomal dominant trait), sex-linked inheritance and hyperactivity of dental lamina. The most accepted theory explains the presence of multiple supernumerary teeth as a result of localized and independent hyperactivity of dental lamina.^8,9^ A combination of environmental and genetic factors also plays a role in the development of supernumerary teeth.^8,9^ In our cases, the exact etiology cannot be related to any hereditary factors as family history was non-contributory and impacted supernumerary teeth were chance findings after radiographic investigations.

In our cases, since the erupted as well as impacted supernumerary premolars were not associated with any complication, the patients were informed about the condition and advised periodic regular follow-ups. Impacted supernumerary teeth, if asymptomatic, can be detected only during radiographic examinations. A complete radiographic survey, including either periapical and occlusal radiograph or panoramic view, is among the most useful radiographic investigations to visualize impacted supernumerary teeth. However, radiographic interpretations of such patients should always be conducted in conjunction with clinical findings to rule out any syndromic involvement. Advanced radiographic techniques such as computed tomography and cone-beam computed tomography are indicated in cases of syndromic manifestations (Table 2)^10-12^ or complications associated with supernumerary teeth. Deepti (2013) proposed a decision-making system for the management of supernumerary teeth. According to this system, impacted or erupted supernumerary teeth without any functional or esthetic complications should be kept on periodic evaluation.^13^

## Conclusion

Supernumerary teeth can be present in any region of the oral cavity. Although the occurrence of maxillary para-premolars is rare, radiographic investigations play a major and decisive role in determining the management of such cases. Hence the clinician should be cautious in case of asymptomatic supernumerary teeth and make appropriate decisions with the help of radiographic aids.

## Acknowledgments

The authors would like to acknowledge Dr. Amogh Tanwar for providing relevant patients data.

## Authors’ contributions

MG and AB performed the radiographic examinations as well as the literature review. RT drafted the manuscript and prepared the figures. VJ, AS, and RH critically revised the manuscript. All authors read and approved the final manuscript.

## Funding

The study was self funded.

## Competing interests

The authors declare no competing interests with regards to the authorship and/or publication of this article.

## Ethics approval

Patients reported in the study have given signed written consents for use of the photographs.
